# Dramatic radiotherapy response of a giant T4 cutaneous squamous cell carcinoma of the scalp with extensive bone destruction: a case report

**DOI:** 10.1186/s13256-021-03213-6

**Published:** 2021-12-25

**Authors:** Isabella Gruber, Oliver Koelbl

**Affiliations:** Department of Radiation Oncology, University Hospital of University Regensburg, Franz-Josef-Strauss-Allee 11, 93053 Regensburg, Germany

**Keywords:** Case report, Cutaneous squamous cell carcinoma, Bone involvement, Head and neck, Radiotherapy, Cemiplimab

## Abstract

**Background:**

Patients with large cutaneous squamous cell carcinoma of the scalp are a treatment challenge. We report a case of dramatic radiotherapy response of a patient with a giant cutaneous squamous cell carcinoma of the scalp with extensive skull destruction and suspected infiltration of the dura mater and superior sagittal sinus. This case is the first report of this kind in the literature that shows that large bone defects can heal with the resolution of tumor and inflammation by secondary intention without surgical reconstruction. We want to put an end to concerns about radiocurability of tumors with extensive bone involvement, and show sustained complete response after definitive radiotherapy and programmed cell death protein-1 inhibiting antibody therapy.

**Case presentation:**

A 74-year-old White man presented with a 7.2 × 6.8 × 5.5 cm painless tumor on the right parietal region of the scalp. Medical imaging revealed widespread destruction of the skull and suspected infiltration of the dura mater and superior sagittal sinus. Biopsies showed cutaneous squamous cell carcinoma (cT4a cN0 cM0, stage IVA). The patient was treated with a total dose of 60 Gy, at 2 Gy per daily fraction with volumetric modulated arc therapy using 6 megavoltage photons. The biologically effective dose (alpha/beta 10 Gy) was 72 Gy. The tumor response correlated with dose received. The patient had a massive tumor necrosis secondary to tumor shrinkage after 18 fractions (36 Gy, biologically effective dose 43.2 Gy). Leakage of cerebrospinal fluid did not occur. Radiotherapy did not hamper the patient’s quality of life. The patient had a clear regression of the initial tumor on the final day of radiotherapy. The bone defect healed by secondary intention without surgical interventions. The patient achieved a complete response with a good cosmetic result after 82 days follow-up. He started a programmed cell death protein-1 inhibiting antibody therapy with cemiplimab 2 months after radiotherapy, and is now at 10 months follow-up without evidence of recurrence.

**Conclusion:**

Definitive radiotherapy is a safe and highly effective therapy for giant tumors of the scalp with extensive bone destruction. We report a sustained complete response with a good cosmetic result after secondary wound healing.

## Background

Giant T4 cutaneous squamous cell carcinoma (cSCC) of the scalp with extensive bone infiltration are a treatment challenge. The subgaleal plane offers little resistance to tumors and cSCC can spread for long distances with invasion of the dura mater, vessels, and brain [1, 2]. In many cases, the size of the T4 cSCC impedes sufficient surgical margins or requires resections with unreasonable cosmetic results [3, 4]. International guidelines consider definitive radiotherapy (RT) as a curative alternative to surgery for patients with cSCC in sensitive anatomical areas of the head and neck where surgery compromises function or cosmesis, and for patients who cannot undergo an operation (for example due to presence of comorbidities). Furthermore, some patients prefer a noninvasive treatment. Definitive RT plays a major role in these patients, with encouraging results [3, 4]. Better understanding of tolerance doses, advances in treatment planning, and better nursing management for patients receiving RT contributes to a low risk of severe late complications after RT, which range from 5% to 9% [5–7]. Unfortunately, there is a misbelief that tumors with extensive bone involvement have worse local control by RT [8]. The literature shows sufficient data to the contrary [5, 6, 8]. Mapping the RT literature of RT demonstrates that cSCC with bone involvement has a 5-year local control of 40% and a 5-year cause-specific survival of 52% after definitive RT [5]. It is time to emphasize the sufficient rates of local control and cause-specific survival after definitive RT. This case must be reported to show that patients with large cSCC of the scalp and massive bone destruction can have a sustained complete response after definitive RT and programmed cell death protein-1 (PD-1) inhibiting antibody therapy. We show the largely unknown fact that even large bone defects can heal with the resolution of tumor and inflammation by secondary intention with good cosmetic results. Given the increase in unresectable tumors, we believe this case has clinical impact across more than one clinical specialty.

## Case presentation

**Fig. 1:**
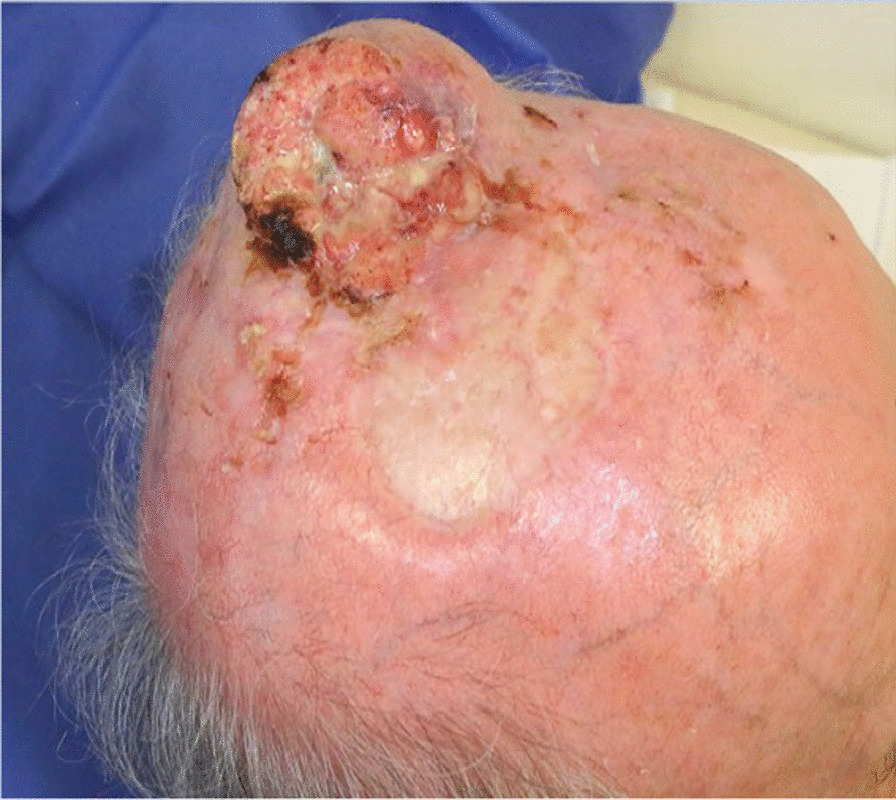
Clinical presentation before definitive radiotherapy. A 74-year-old White man presented to our emergency department with a 7 × 6 × 4 cm tumor on the right parietal region of the scalp. The patient was conscious and painless.

**Fig. 2:**
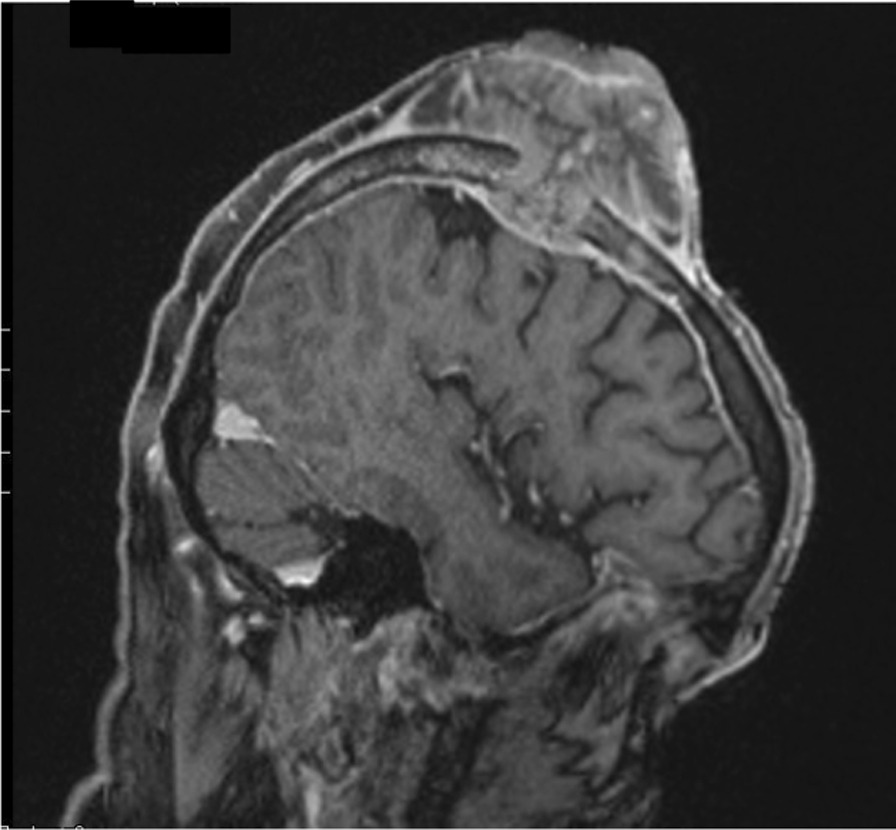
T1 MP-RAGE 3D weighted sagittal contrast-enhanced MRI sequence before definitive radiotherapy. MRI showed a 7.2 × 6.8 × 5.5 cm tumor with destruction of the skull and suspected infiltration of the dura mater and superior sagittal sinus. Biopsies showed cutaneous squamous cell carcinoma. The TNM stage was cT4a cN0 cM0, stage IVA using the Union for International Cancer Control (UICC) staging system (8th edition).

**Fig. 3:**
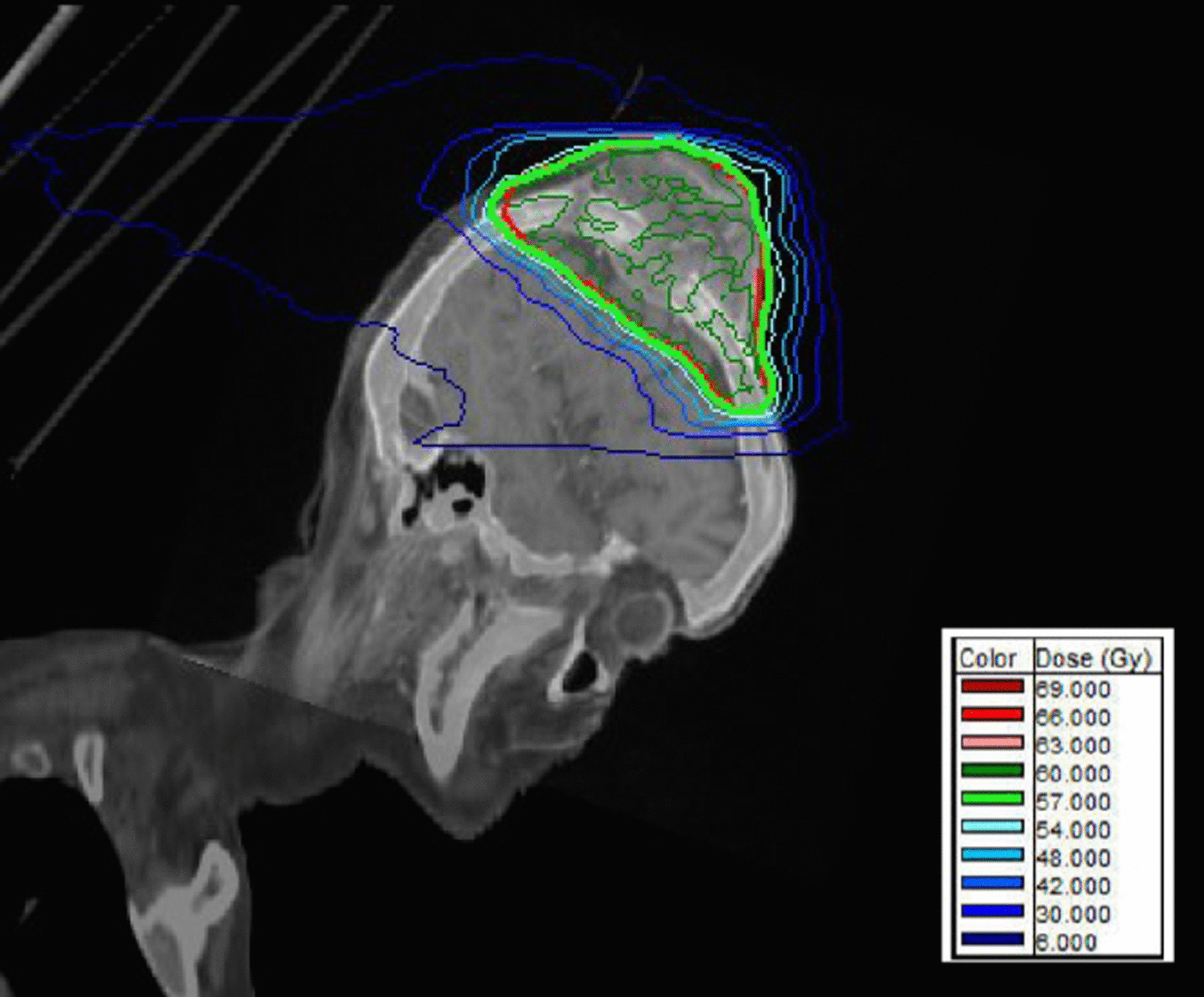
Sagittal view of planning computed tomography images in fusion with MRI. The planning target volume (thick red line) covered the gross tumor volume plus an appropriate margin and was treated with a total dose of 60 Gy at 2 Gy per daily fraction over 6 weeks (BED _10_ 72 Gy). Radiation dose distribution represented by radiation isodose lines: The thick red line represents the planning target volume and the thick green line represents the 57 Gy isodose line (that is 95% isodose, encompassing planning target volume). One cm thick bolus material was used to ensure sufficient surface dose.

A 74-year-old White man presented to the emergency department of our institution with a 7 × 6 × 4 cm tumor on the right parietal region of the scalp. Figure 1 shows the patient at the time of presentation. The patient was conscious and painless. He suffered from psoriasis vulgaris and advanced psoriasis arthropathy, with contracture of the shoulders and gibbus of the thoracic spine. He gave a history of immunosuppression and intense ultraviolet exposure without a family history of cancer. The patient reported that the tumor had begun as a small lesion approximately 5 months prior, and continued to expand to its measured size at presentation. The patient record showed that the patient had a cSCC in the middle of the scalp 6 years ago, which was resected with a narrow resection margin of 6.8 mm and covered with a split skin graft of the right thigh (pT2 cN0 cM0, stage II). A new histological confirmation of the recurrent cSCC was obtained. The patient had a clinically negative neck without evidence of metastatic spread. Magnetic resonance imaging (MRI) showed a 7.2 × 6.8 × 5.5 cm tumor with widespread destruction of the skull and suspected infiltration of the dura mater and superior sagittal sinus (Fig. 2). Fluorine‐18‐fluorodeoxyglucose positron emission tomography-computed tomography (18 F-FDG-PET-CT) revealed the known tumor of the skull. The TNM stage was cT4a cN0 cM0, stage IVA using the Union for International Cancer Control (UICC) staging system (8th edition). There was no useful operative option. Definitive RT was recommended. The patient underwent planning computed tomography (SOMATOM Sensation open of Siemens AG Medical Solutions). He was simulated for RT in a reproducible position with lifted upper body. A thermoplastic head mask was used for immobilization and fixation. A bolus material was placed over the tumor to ensure sufficient surface dose. Monaco external beam-planning software (Version 5.11, Elekta) was used for contouring and volumetric modulated arc therapy (VMAT) planning. Figure 3 shows CT planning sagittal slices through the tumor with isodoses from the original RT treatment plan. The planning target volume (PTV) covered the gross tumor volume plus an appropriate margin, and he was treated with a total dose of 60 Gy at 2 Gy per daily fraction over 6 weeks (biologically effective dose; BED _10_ 72 Gy). We performed VMAT at Elekta Synergy Agility Linear Accelerator with a 160 multileaf collimator by 6 megavoltage (MV) photons and a dose rate of daily 478.77 monitor units. The physical treatment plan consisted of two arcs.

**Fig. 4:**
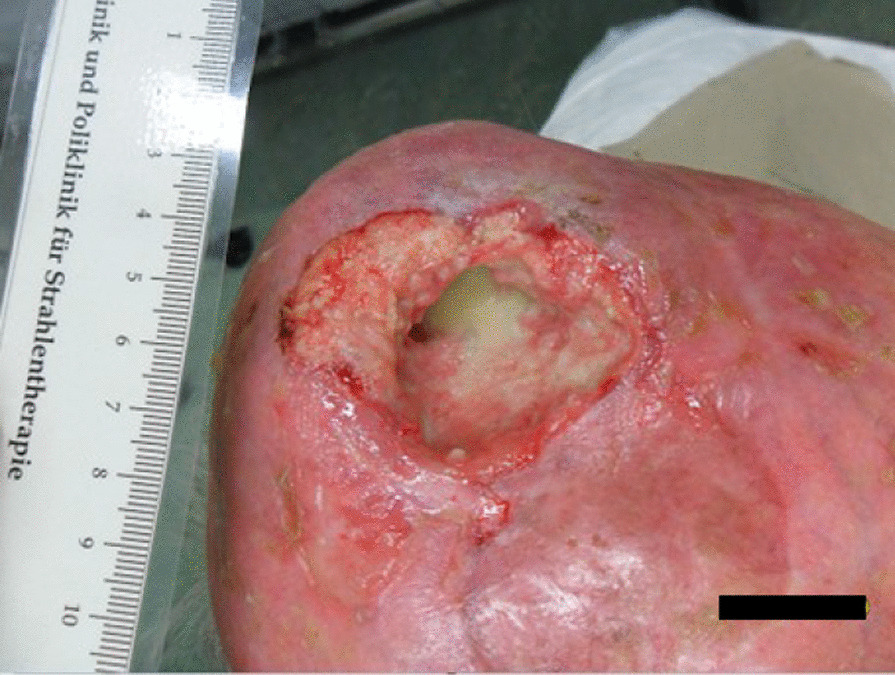
Clinical appearance after 36 Gy (BED _10_ 43.2 Gy). We found rapid tumor shrinkage and visible dura mater on the base of the ulcer after 18 fractions. Acute side effects included radiation-induced dermatitis CTC grade 3 using the Common Terminology Criteria for Adverse Events (CTCAE v. 4.0) and spotty bleeding. Leakage of cerebrospinal fluid did not occur.

**Fig. 5:**
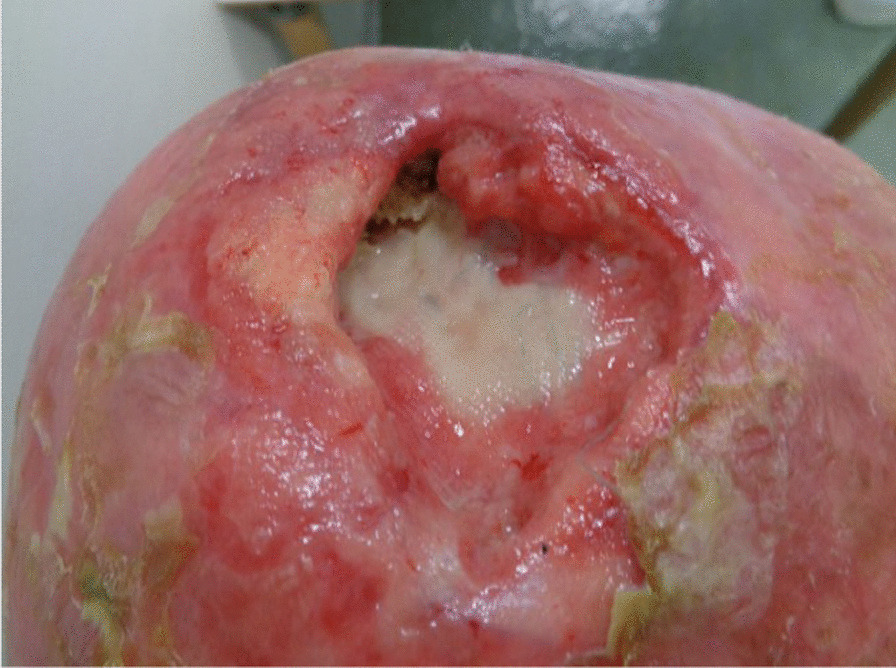
Clinical appearance on the final day of radiotherapy (60 Gy, BED _10_ 72 Gy). The patient had a clear regression of the initial tumor. Intense nursing care contributed to favorable safety and tolerability of definitive radiotherapy.

**Fig. 6:**
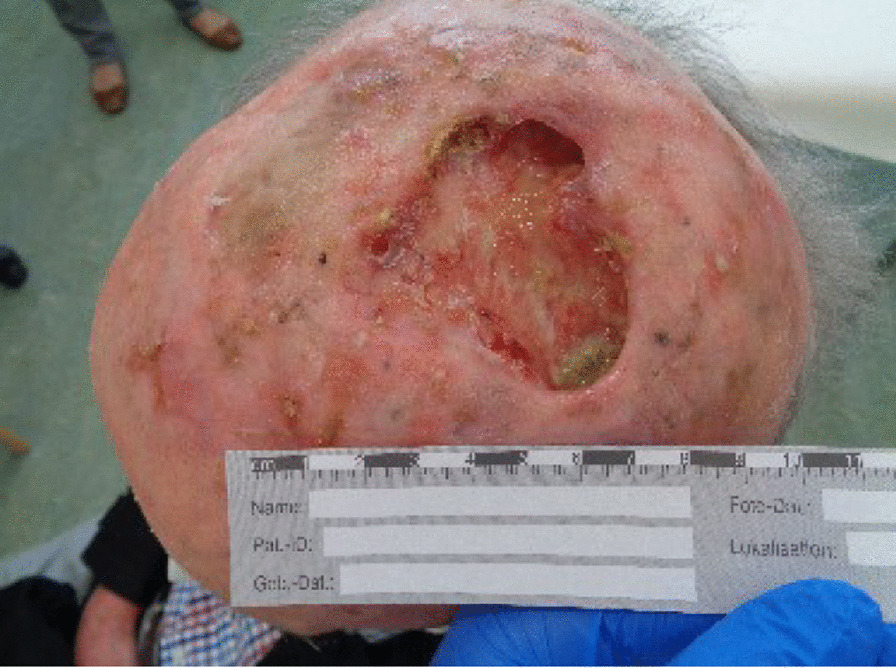
Clinical appearance 82 days after definitive radiotherapy. The patient was alive with no evidence of disease. The defect of the scalp healed rapidly with the resolution of tumor and inflammation by secondary intention without infection or new tissue breakdown. The patient did not need surgical or reconstructive interventions.

**Fig. 7:**
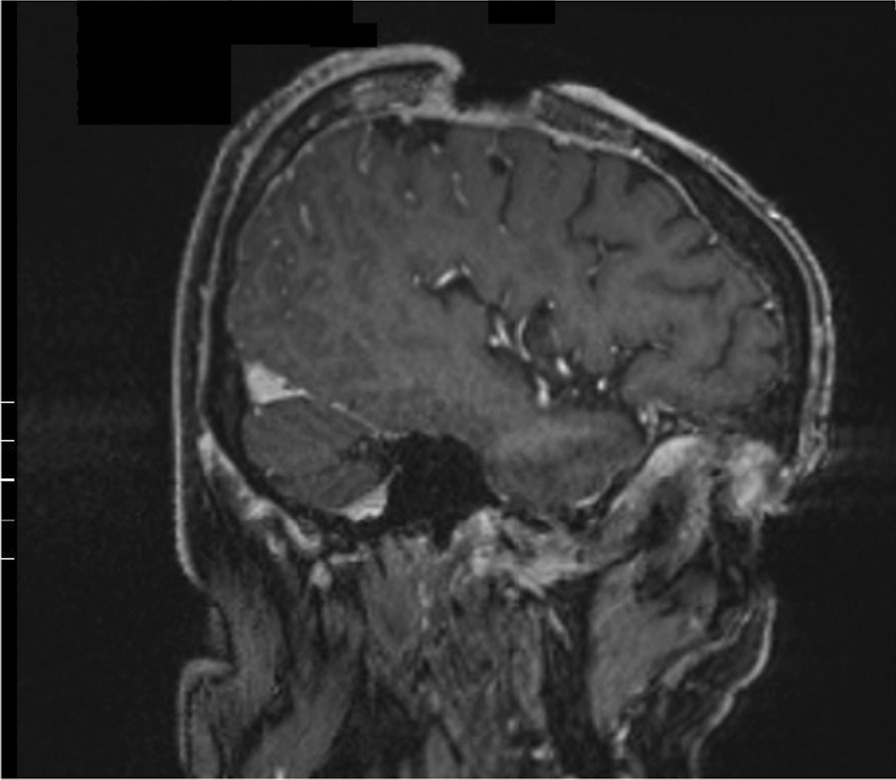
T1 MP-RAGE 3D weighted sagittal contrast-enhanced MRI sequence 3 months after radiotherapy. MR image showed a dramatic response to definitive radiotherapy with residual inflammatory tissue on the base of the initial ulcer.

**Fig. 8:**
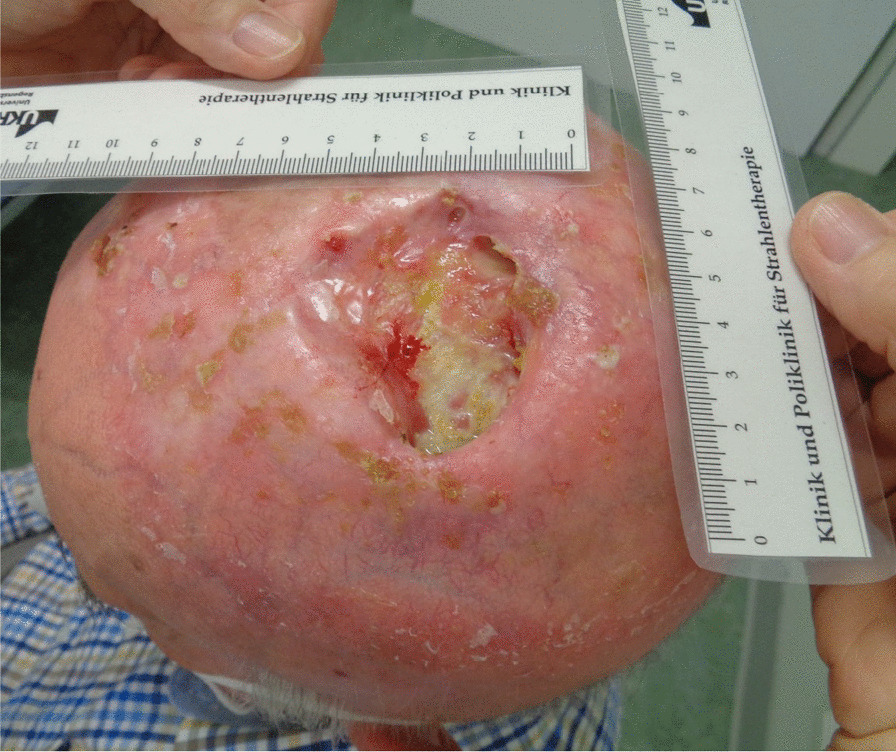
Clinical appearance at 10 months follow-up during PD-1 inhibiting antibody therapy with cemiplimab. The patient presented with a good cosmetic result without evidence of tumor recurrence. He had no late complications after radiotherapy. Treatment with cemiplimab was well tolerated without adverse events.

The tumor response correlated with the dose received. The patient had a massive tumor necrosis secondary to rapid tumor shrinkage after 18 fractions (36 Gy, BED _10_ 43.2 Gy). We found visible dura mater on the base of the ulcer. There was no leakage of cerebrospinal fluid (Fig. 4). Acute side effects included radiation-induced dermatitis CTC grade 3 using the Common Terminology Criteria for Adverse Events (CTCAE v. 4.0) and spotty bleeding. Daily visits and nursing care were provided to prevent infection. The patient had a clear regression of the initial tumor on the final day of RT (60 Gy, BED _10_ 72 Gy). The base of the necrosis appeared mainly to comprise granulation tissue (Fig. 5). The patient was painless at any point in time. He did not require treatment breaks for complications. Granulation and reepithelialization proceeded from the wound edges within 10 days after RT. The large wound healed rapidly with the resolution of the tumor and inflammation by secondary intention without infection or new tissue breakdown. We found no late complications. The patient did not need surgical or reconstructive interventions. RT permitted a clinical complete remission at 82 days follow-up (Fig. 6). MR images obtained 3 months after irradiation showed residual inflammatory tissue on the base of the initial ulcer (Fig. 7). The patient started a PD-1 inhibiting antibody therapy with cemiplimab 350 mg every 3 weeks, administered as an infusion 2 months after the last RT. The treatment with cemiplimab was well tolerated without adverse events. The patient is now at 10 months follow-up without clinical evidence of tumor recurrence and continues therapy with cemiplimab (Fig. 8).

## Discussion

This case is important to medicine because it shows a dramatic response to definitive RT. Extensive tumor shrinkage led to clearly visible dura mater and spotty bleeding without bigger hemorrhage. Massive tumor shrinkage can damage cerebral blood vessels and result in a breakdown of the blood–brain barrier. Fortunately, our patient had no leakage of cerebrospinal fluid or wound infections. Intense nursing care contributed to favorable safety and tolerability of definitive RT. No further surgical interventions were required. This report raises the possibility that tumors with extensive soft tissue and bone involvement can successfully be treated with definitive RT. RT did not hamper the patient’s quality of life. The defect of the scalp healed by secondary intention. The literature shows that wound healing by secondary intention can successfully be used for defects of the scalp in patients who are poor candidates for surgical reconstruction [9]. The illustrated patient was highly satisfied with the treatment and the excellent outcome of definitive RT. It will be interesting to document patients’ perspective and quality of life with the patient-reported outcome measures at specific time points during and after treatment of advanced skin tumors. Disease-free survival and overall survival are reasonable metrics but do not analyze the experience of patients during and after treatment [10].

Our patient was treated with conventional fractionation (2 Gy/fx with BED _10_ 72 Gy) in accordance with international recommendations [3, 4]. The American Society for Radiation Oncology published an upgraded guideline for definitive RT for cSCC in 2020 [3]. Definitive RT is recommended as a curative treatment in patients with cSCC who cannot undergo or decline surgical resection (strong strength of recommendation and moderate quality of evidence) [3]. Definitive RT is conditionally recommended as a curative treatment modality in patients with cSCC in anatomical areas where surgery can compromise function or cosmesis (conditional strength of recommendation with moderate quality of evidence) [3]. The reason for this weak level of evidence is the lack of prospective randomized controlled trials comparing the effectiveness of definitive RT in patient survival and local tumor control to other local therapies [3]. Pending prospective data, retrospective studies show durable local tumor control and functional and cosmetic preservation by definitive RT [5–7, 11].

Our patient had an unsuccessful surgical treatment 6 years ago. This is associated with a high level of risk because recurrent tumors show poor local tumor control by RT [5–8, 12]. Lee *et al.* [5] conducted a multivariate analysis of definitive RT for 68 stage T4 tumors of the skin of the head and neck, and analyzed local control and cause-specific survival rates of definitive RT as a function of previous treatment. The 5-year initial local control rate of previously untreated tumors was 67% and of recurrent tumors 41% (*p* = 0.07) after RT. The cause-specific 5-year survival differed significantly between previously untreated and recurrent tumors after RT (93% versus 58%, *p* = 0.003) [5]. This stresses the importance of sufficient initial treatment of cSCC for optimal local control and survival by RT.

The definitive RT of advanced T4 cSCC of the head and neck shows 5-year local control rates of 50–60% [5–7, 12]. It is a popular misbelief that definitive RT cannot lead to local control and cure when tumors infiltrate bone [8]. The literature shows enough data to the contrary, although cSCC with bone involvement show a lower probability of cure by RT [5, 6, 8]. Lee *et al.* [5] showed results of definitive RT of tumors invading bone. The involvement of bone decreased the probability of initial local control (62% versus 40%, *p* = 0.08) and cause-specific survival by RT at 5 years (92% versus 52%, *p* = 0.001) [5]. In summary, we must note, that definitive RT leads to a relatively high rate of local control and cause-specific survival for this unfavorable group of tumors with infiltration of bone.

## Conclusions

We report a remarkable sustained response to definitive RT in an elderly patient with a giant tumor of the scalp. Modern treatment planning and intense nursing care contributed to a good cosmetic result after secondary wound healing. Definitive RT achieves high local control rates without severe acute or late side effects. The currently available literature lacks prospective randomized trials for the use of definitive RT of tumors with extensive bone infiltration, but the presented case report shows both favorable safety and tolerability and a high effectiveness of definitive RT.

## Data Availability

All data generated or analyzed during this study are included in this published article.

## Abbreviations

cSCC: Cutaneous squamous cell carcinoma
RT: Radiotherapy
MRI: Magnetic resonance imaging
18 F-FDG-PET-CT: Fluorine‐18‐fluorodeoxyglucose positron emission tomography computed tomography
UICC: Union for International Cancer Control
VMAT: Volumetric modulated arc therapy
PTV: Planning target volume
MV: Megavoltage
BED: Biologically effective dose
CTCAE: Common Terminology Criteria for Adverse Events
